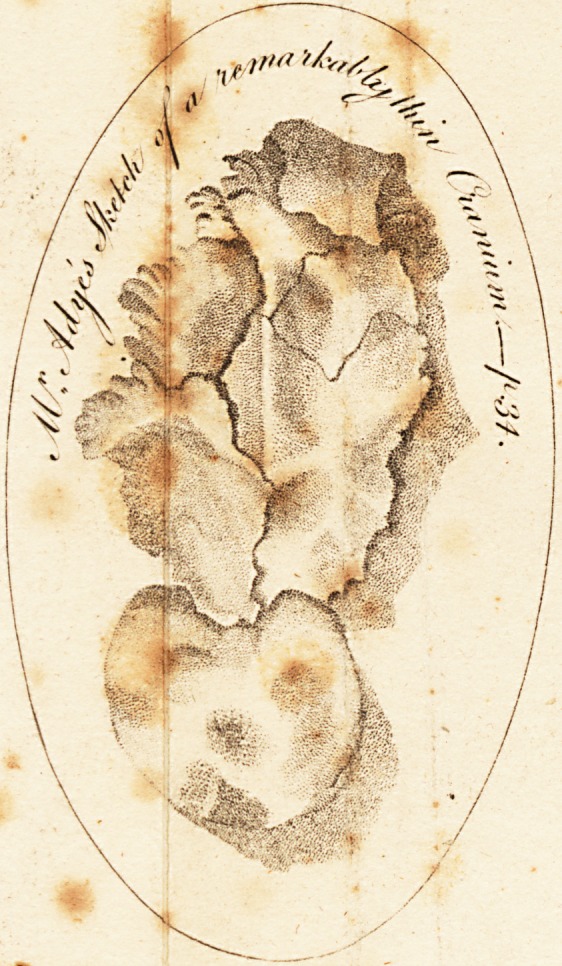# Mr. Adye's Case of Fractured Scull

**Published:** 1804-01-01

**Authors:** W. F. Adye

**Affiliations:** Bradford, Wilts


### *To the Editors of the Medical and Physical Journal.*

GENTLEMEN,

IN reading your Medical and Physical Journal, No. 55, I noticed Mr. Smith's sketch of an extraordinary thick cranium; and having lately had a patient with a fractured scull, (*see plate*) where the bone was as remarkably thin, and the case altogether not a very common one, I beg leave to present you with it for publication. I have also enclosed the bones, joined together with wire.

I am, &c.

*Bradford, Wilts,*

*Oct.* 9, 1803.

W. F. ADYE.

---

WM. CROMWELL, aged 17, a pauper of this parish, on the 1st of August last, received a blow from the end of a trap-stick, trap-stick, upon the os frontis, about two inches above the orbit of the left eye, which produced a fracture, with considerable depression.

The next morning the trephine was applied upon the superior part of the sound bone, and five depressed pieces of remarkably thin bone, (including a large portion of the orbit) were removed; a considerable hæmorrhagy attended the operation, proceeding from a wound of the brain, immediately under the most depressed part; this however subsided after a short time, by moderate pressure.

On the fourth day a fungus arose from the brain, increasing to the size of a large watch in the course of six days, having an opening in the middle, from whence brain was regularly discharged for nearly a week. Various astringents having been employed for some time, with very little effect, recourse was had to strong pressure, which gradually removed it, without producing any alarming symptoms.

During the first six weeks, the patient was unable to open his left eye without shutting the right; but is now recovered in every respect.—The wound was quite healed, and the boy returned to his usual occupation, (that of a weaver) the first week in October.

## Figures and Tables

**Figure f1:**